# Progress on quality management in the German health system – a long and winding road

**DOI:** 10.1186/1478-4505-5-7

**Published:** 2007-06-05

**Authors:** Juergen Breckenkamp, Christiane Wiskow, Ulrich Laaser

**Affiliations:** 1 University of Bielefeld, School of Public Health, Bielefeld, Germany; 2 Salumondi, International Public Health, Geneva, Switzerland; 3 Institute for Scientific Analysis in Health Care (IWAG), Bielefeld, Germany

## Abstract

The interest in quality management in health care has increased in the last decades as the financial crises in most health systems generated the need for solutions to contain costs while maintaining quality of care. In Germany the development of quality management procedures has been closely linked with health care reforms. Starting in the early nineties quality management issues gained momentum in reform legislation only 10 years later.

This review summarizes recent developments in medical quality management as related to the federal reform legislation in Germany. It provides an overview on the infrastructure, actors and on the current discussion concerning quality management in medical care.

Germany had to catch up on implementing quality management in the health system compared to other countries. Considerable progress has been made, however, it is recognized that the full integration of quality management will require long-term commitment in developing methods, instruments and communication procedures. The most ambitious project at present is the development of a comprehensive comparative quality management system for hospitals at national level, including public reporting. For the time being medical quality management in Germany is dealt with as a technical and professional issue while the aspects of patient orientation and transparency need further advancement.

## Background

Medical care in Germany is recognized as having a high standard in general. The health care is mainly financed by a fee-based system with a multiplicity of health insurances. The German system known as Bismarck model is the oldest health insurance system in the world, established in 1883. It is still characterized by obligatory insurance, a large number of group specific insurances, guaranty of continued salary payments in case of prolonged incapacity, and division of fees between labourers and employers. Although modified in many details the basic structures of the system have survived since. Nevertheless the Bismarckian systems reached the limits during the nineties mainly because of technological advances in health care, demographic ageing, and increasing unemployment. The crisis of health care financing lead to reform processes which also included the issue of quality assurance.

Quality assurance is an integrated part of health professions with a long tradition of developing methods to assess the quality of work [1]. Generally, quality assurance measures aim at maintaining agreed upon standards, quality management consists in prospective strategies for improving quality [2]. In health systems quality strategies aim at optimizing the provision of health care by identifying inadequate delivery of care and ultimately to influence positively the population morbidity and mortality [3].

This review summarizes recent developments in medical quality management related to the federal reform legislation in Germany. It draws a map of the present institutional actors and infrastructure, and touches on the related issues of medical errors and patient orientation. It provides an overview of the state and the current discussion concerning quality management in medical care. The information is based on a report prepared in 2003, which included expert interviews [4], and has been updated by a review of the recent literature.

### Quality management in legislation and health care reforms

The regulation of health benefits and medical services provided within the statutory health insurance system (GKV, Gesetzliche Krankenversicherung) is laid down in the Social Welfare Code, volume V (SGB V, Sozialgesetzbuch V). It stipulates the regulation, registration, accreditation and control of health service providers within the GKV funded services. The paragraphs 135 – 139 of SGB V are specifically addressing the quality assurance matters in medical care. One characteristic of the German health system is the tradition of self government of health care providers, namely the representative bodies of physicians and health insurance funds. Accordingly it is a principle to make providers and insurances responsible for the implementation of quality assurance measures including the necessary financing. Another special feature of the German health system is the administrative division of services in two sectors, the in-patient care and the ambulatory care. It is meanwhile recognized that lacking coordination at the interface of the two sectors affects the continuity and thus the quality of care and health outcomes for patients. This problem has been started on by a stepwise introduction of disease management programmes, integrative care concepts and inter-sectoral forms of institutions in several reforms of the health system which were based on the respective legislative changes.

The origin of the German health care reform legislation in the nineties lies with the urgent need to control (and to lower) the costs of health care. Within these reforms quality management of medical care was accorded a more prominent role, mainly discussed under the aspect of cost effectiveness. Obligatory quality assurance measures for providers, especially hospitals, within the statutory health insurance system (GKV) entered legislation in 1992 with the law on reforming the health system structure (GSG, Gesundheitsstrukturgesetz,) which came into force in January 1993. Several adjustments of the health system in the period between 1993 and 2000 focussed exclusively on cost reduction.

While the GRG 2000 also emphasized cost containment, however with limited effect, several of its provisions have had effects on the quality aspects of service delivery, such as:

1) the obligatory introduction and development of internal quality management systems in hospitals,

2) inter-sectoral contracts between providers and insurance funds (Sektorübergreifende Versorgungsverträge),

3) the introduction of an optional family doctor model (Hausarztmodell) and financial incentives for patients staying with the same general practitioner as their family doctor to achieve better informed and therefore more cost-effective decision making,

4) the introduction of a restrictive list of effective drugs (Positive List) replacing the concept of a list of ineffective drugs on the market (Negative List).

The Diagnoses Related Groups (DRG) concept has been introduced in hospital care. Steps towards a comprehensive system of quality management were undertaken through cost-benefit-analysis of medical technologies and through the development of treatment guidelines. The improvement of patient rights and patient protection was targeted through financial support of consumer associations and mandating the medical services of the statutory health insurers to evaluate the causes of medical accidents.

These effects on quality management have been enhanced with the latest reform of the health care legislation, laid down in the act on the modernization of the health care system (GMG 2003, Gesundheitsmodernisierungsgesetz). The GMG was adopted in 2003 and entered into force in January 2004. While the GMG 2003 also intends to contain the costs of health care, the Ministry of Health and Social Security (BMGS) stated that the improvement of health care quality is one of the main goals of the reform [5]. Indeed, obligatory quality management requirements have been extended for providers, and the restructuring of the regulatory bodies aims at better coordination at federal and state levels. Cornerstones of this reform act with regard to quality management are:

○ The obligation of the office based practitioners to introduce internal quality management,

○ The foundation of a national institute for quality and economy in the health sector,

○ The restructuring of the federal bodies mandated for regulating licensing and quality management,

○ The introduction of a patient representative at federal level and patient empowerment measures, such as the consultation right of patient representatives in decision making bodies.

The DRG based payment schemes have become mandatory since 2004 and this change in practice has implied an increased need for quality management [6].

### Actors in quality management

There is a broad range of actors regarding quality management in the German health sector. Changes in the structure of institutional actors and their responsibilities constantly occurred with nearly each reform act. We describe below the present major institutional actors as related to the latest health legislation without being exhaustive and complete.

In January 2004 the Federal Joint Committee (G-BA, Gemeinsamer Bundesausschuss) has been institutionalised as a legal entity under public law. The G-BA replaces several former regulatory bodies, it has been jointly formed by the federal association of GKV contracted physicians (KBV, Kassenärztliche Bundesvereinigung), the German Hospital Federation (DKG, Deutsche Krankenhausgesellschaft), and the federal associations of health insurance funds.

The G-BA is the highest decision making body in the joint self-governing structure, reporting to the Ministry of Health (BMGS). Main responsibilities of the G-BA relate to the assessment and licensing of new methods of examination and treatment, particularly in ambulatory care, the development and issuing of directives in medical care, and the regulation of medicament remuneration under GKV approval [7].

Further, a range of responsibilities related to quality management in the health system have been assigned to the G-BA. These include:

1) the development and implementation of directives and criteria for quality management in ambulatory medical and dental care,

2) the decision on quality assurance measures in GKV accredited hospitals,

3) financial sanctions for hospitals not complying with quality management directives,

4) content and volume of the quality reports to be issued by the hospitals.

Since 2004, the decision making process with regard to quality management in hospitals requires the consultation with the Federal Medical Chamber (BAEK, Bundesärztekammer), the Federation of Private Health Insurance Funds, and the Nursing Association. These organizations participate in the negotiations but have no vote [8].

The composition of the G-BA plenary includes 3 impartial members, 9 representatives of health insurance funds and 9 representatives of providers. The compositions of the various sub-committees vary according to the relevance of decisions to be taken. Additionally up to 9 patient representatives have the right to participate in the committees, but without vote [7].

Appointed by the G-BA as the centre for quality management in hospitals is the Federal Office for Quality Assurance (BQS, Bundesgeschäftsstelle Qualitätssicherung), an independent service agency. BQS tasks focus on the external comparative quality management which is obligatory for hospitals accredited under GKV. The BQS was launched in 2001 and since then is coordinating the processes for obtaining and providing the benchmarking information for hospitals. All German hospitals are participating in the external QA procedures organised along federal and state based offices. In the transition phase from fee-based remuneration to DRGs, the BQS provided support.

The Institute for Quality and Economy in Health Care (IQWiG, Institut für Qualität und Wirtschaftlichkeit im Gesundheitswesen) has been founded in 2004 as an independent institution. The institute name is programme. Major tasks assigned by the G-BA and the Ministry of Health include the scientific outcome analysis of medical treatments and drugs, the evaluation of treatment guidelines, and the development of recommendations regarding disease management programmes. Additionally the institute is responsible for providing information to patients and the public [9].

The Working Group for Promoting Quality Assurance in Medicine (AQS, Arbeitsgemeinschaft zur Förderung der Qualitätssicherung in der Medizin) is another body based on the SGB V legislation. Its core tasks are the system wide evaluation of quality management in Germany and the development of recommendations for a coherent and comprehensive quality management system across sectors and professional groups.

Other major institutional actors in medical quality management include the German Institute for Medical Documentation and Information (DIMDI, Deutsches Institut für Medizinische Dokumentation und Information), the Central Agency for Quality in Medicine (AeZQ, Aerztliches Zentrum für Qualität in der Medizin), the German Guideline Clearing House (Deutsches Leitlinien Clearingverfahren), the Association for Quality Management in Health Care (GQMG, Gesellschaft für Qualitätsmanagement in der Gesundheitsversorgung), the German Network of Evidence Based Medicine (DNEbM, Deutsches Netzwerk Evidenzbasierte Medizin), and the Association of the Scientific Medical Societies in Germany (AWMF, Arbeitsgemeinschaft der Wissenschaftlichen Medizinischen Fachgesellschaften). In the area of nursing the German Network for Quality Development in Nursing (DNQP, Deutsches Netzwerk fuer Qualitätsentwicklung in der Pflege) should be mentioned.

### Structure of quality management

#### Ambulatory medical care

As stipulated in the GMG 2003 the providers of ambulatory medical care (in Germany mostly office-based physicians) are obliged to introduce internal quality management systems additionally to the participation in external quality assurance measures. The details are not laid down in the legislation. The G-BA was assigned to develop the respective guidance. It is expected that the choice of quality management systems will be left to the providers as the individual local situation will determine the appropriateness of the scope of quality management [10]. Concerning external quality management, it is in the responsibility of the associations of GKV accredited physicians (KV) to conduct the monitoring and control, on a sampling basis in the frame of G-BA guidelines. The obligation to structured continuing education for physicians is one feature of quality management in the GMG 2003. Quality circles for office based physicians have been introduced in the past years as one key element. Currently there are approximately 3500 quality circles operating in Germany [5].

#### Hospital care

For the obligatory external comparative quality management in hospitals the G-BA has formed a sub – committee, which advises the G-BA and cooperates with the technical working groups at federal level as well as with the steering committees at state level. All data are gathered via the quality offices at state level (LQS), and analysed and published by the BQS. In each state (Land) a steering committee, in close cooperation with the LQS and BQS, is responsible for the implementation of quality management in their region. Independent technical working groups dialogue and collaborate directly with the hospitals in supporting them to work with the results of the audit and to sustain quality management continuously. Regional quality conferences and the so-called structured dialogue are means for this cooperation [8].

**Figure 1:**
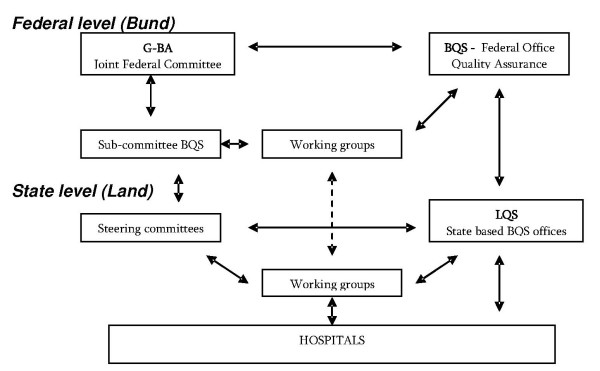
**The structure of external quality management in hospitals**. (source: translated from BQS)

Figure 1 provides an overview on the infrastructure.

### Quality initiatives

The implementation of the legislation and the way how different stakeholders translate it into practical initiatives of quality management is illustrated by describing some selective examples.

*The Cooperation for Transparency and Quality in Hospitals *(KTQ^®^, Kooperation für Transparenz und Qualität im Krankenhaus) was initiated in 1997 by the federal chamber of physicians (BAeK) and the federal associations of the various health insurance funds. It was later joined by the hospital federation and the nursing council. The KTQ mission is to strengthen transparency in the performance of hospitals by providing comprehensible information to all concerned, including patients [11,3]. Critique on the method was mentioned in stating the KTQ mainly focuses on structural quality, while other quality management systems meanwhile had a broader approach [12].

The *Pilot Project Quality Management in Hospitals *(DemoProQM, Demonstrationsprojekt Qualitätsmanagement im Krankenhaus) was initiated and funded by the Ministry of Health. The aim of the project was to demonstrate the benefits of quality management for providers, insurance funds, and patients. Between 1998 and 2001 the project supported 44 hospitals in introducing comprehensive quality management systems, which are patient-and employee-oriented as well as interdisciplinary and covering all hierarchical levels [3].

The project *Hospital Quality Model *(QMK, Qualitätsmodell Krankenhaus) focussed on the development of instruments for measuring and comparing the aspect of outcome quality in hospitals. For this purpose a voluntary cooperation between the Federation of Local Health Insurance Funds (AOK Bundesverband) and 20 hospitals was initiated in 1999. The project aimed at defining valid and reliable outcome indicators and developing practice-relevant instruments. It followed a comprehensive and innovative approach by including the perspective of patients and referring physicians in their instruments. The results of the project have been recognised as a valuable contribution to the development of internal quality management in hospitals and the instruments were considered being of use for external quality comparisons as well [13].

With *Quality and Development for Practice (*QEP^®^, Qualität und Entwicklung in Praxen) a specialised quality management system for GKV accredited physicians and psychotherapists in ambulatory care has been developed by the KBV. The method is indicator based and addresses the issues patient safety and care, information and documentation, collaborators, office organization and basic conditions, and continuing education. Since March 2004 QEP is in the pilot testing phase in 60 practices, it is due to external evaluation since summer 2005 with the aim of approval as a routine quality management system in ambulatory practice [14].

### The state of external quality management in hospitals

The external comparative quality management for hospitals is a highly discussed subject in the German health community. The legislation and the relevant bodies have chosen a stepwise approach for introducing the external comparative quality management. Since 2001 yearly nationwide reports on quality in hospitals have been published by BQS. The latest report 2003 for the first time contained the complete results. Further, data analysis has been carried out anonymously providing for a kind of "protected" comparison, aimed at preventing stigmatisation of hospitals. It reveals a prevailing concern of misinterpretation of quality information by the public. [15]

In November 2004 the yearly outcome conference on the external quality management was organized for the first time by the newly established Joint Federal Committee, G-BA. The Committee stated that in no other country a similar project to develop a comprehensive system of comparative hospital quality data exists [16]. While in general the conference drew a positive picture in highlighting the high quality standard of hospital care and the progresses made concerning the development and delivery of quality data in the past years, the critical points were reflected in the statements of the major stakeholders.

It was recognized that the current scope of quality data had to be seen as a valuable beginning with the aim to continuously extend the coverage further. The current quality data covered 33 performance areas, such as orthopaedics, some surgical areas, cardiology, breast-cancer treatment and nursing, representing 20 per cent of the overall services.

One of the challenges was to transfer the obtained data and results into practical quality improvement activities. Each hospital received its individual results in comparison to other anonymous hospitals. One quarter of the cases were notified on conspicuous results. In reporting back to the queries, some hospitals could clarify confounders or poor documentation, others investigated in the causes and improved work procedures subsequently [17]. Where no satisfactory feedback from the notified hospitals was received, other measures of the structured dialogue were activated, such as consultative meetings or on-site visits. The aim of the structured dialogue was to advise and support the hospitals in improvement activities targeting the problematic areas. In cases of non-cooperation or non-compliance of a hospital, financial sanctions were possible in reduction of compensation parts or – in extreme cases – the termination of the provider contract within GKV. In general the development of comparative quality management has been considered being on a good way, however the instruments needed to be improved and, especially the bureaucracy of data generation and communication for the hospitals had to be reduced significantly in order to assure reliable data gathering [18].

Nursing features were included in the quality data sets since 2002. From a nursing perspective it was suggested that indicators should be directly relevant for patients and the development of more cross-sectional general indicators such as falls and pain management. General indicators were therefore recommendable in future for achieving better patient orientation [19].

From the viewpoint of the patient representatives a critical aspect was the highly technical level of results and the report was therefore not of use for patients. They suggested developing additional presentation options appropriate for the target audience of patients, and establishing information offices for their referring general practitioners. Problematic was the fact that currently the results were anonymous which represented an impediment to the transparency of hospital performance for their users [20].

The lack of transparency regarding the quality of hospital performance is a common critique. Because of the anonymous data a comparison of quality is only possible for the hospitals themselves. Currently no external user could identify from the BQS report the quality of a specific hospital. Hence, the report was of no use for patients, and neither the office based practitioners could fulfil their task in recommending hospitals to their patients [15]. Germany has been classified a developing country in public quality reporting compared to other countries and it was noticed that high expectations of the public and the patients were facing the reservations of interest groups in the health sector [21,6]. The classical quality assurance approach reflected rather an intra-professional control system which guaranteed minimum standards in all hospitals but would not necessarily have to be transparent to the public [12]. Despite considerable and expensive efforts the transparency of hospital quality and outcomes remains insufficient [22,12].

In general it was noted that the current emphasis on structure and process quality was not sufficient and an enhanced orientation towards outcome quality would be necessary [23]. The current lack of outcome quality indicators has been associated with methodological limitations of hospital self-reporting, such as the challenge of inter-hospital risk adjustment. The limited time frame for observations in hospital settings was another obstacle, as the result of a hospital treatment often could only be measured in the post-hospital period [12].

The quality of the data is a major methodological challenge in the external quality management development. It was argued that methods for validating quality indicators have been imported from international concepts without evaluating their effectiveness in the German context [24]. Some of the quality indicators, such as the volume-outcome-relationship, have been discussed controversially [25,21,26]. Following critics on the reliability and validity of data, a data quality monitoring system has been developed with controls on a sampling basis [18].

Regarding the evaluation of quality assurance and management processes in Germany progress has been made, but deficits are still observed [27]. An international review of hospital quality strategies revealed that there was in general only little research assessing the effectiveness of hospital or national quality strategies. Thus no strong evidence on the effectiveness of strategies could be found, though this would not mean that they are not effective. Publications seemed to be biased towards success stories rather than failures [28].

#### Quality report 2005

From 2005 onwards hospitals have to submit a quality report every two years to the health insurance funds. The structure and content of the reports are framed by the self-governing partners through G-BA. As stipulated in the GMG 2003 the reports have to be published and be accessible for the public. This is a further step towards more transparency of health care quality. However, several critical remarks point to the limitation of these reports for the time being. One argument was the lack of outcome quality results in the 2005 reports, which was related to methodological problems as mentioned above [12]. The GQMG noticed that there was not yet an agreed definition of a quality report and therefore discussed the definition of quality indicators. At the same time it welcomed the increased consideration of outcome quality in the quality reports and underscored the need to address indicator validity and reliability issues [29]. The two year interval for the quality reports was considered too long to provide up-to-date information [29,30]. A target conflict of the reports additionally limited their relevance for external users: while quality reports proved to be a useful instrument for internal quality management, the purpose of creating more quality transparency for users was not assured. It was assumed that the hospitals would use this part of the report rather for marketing purposes. Because of the extensive application of technical terms the use of the reports for patients was questioned [30]. Further, evidence from other countries with long experience in public quality reports suggests that the reports were in general not used for decision making, misunderstood by the public or the information was mistrusted. Nevertheless, consumers and general public supported the principle of public quality reporting. The experiences showed that imposed reports on unwilling professionals and a reserved public would not work. Rather a partnership between the parties concerned would be favourable for successful implementation [31]. The controversial discussion of the quality reports in Germany reflects these aspects. However, the involvement of the major stakeholders in deciding on the quality reports through the G-BA structure should be seen as a factor conducive for the future development and use of this instrument. While the reports were considered as a step in the right direction, it was recommended to stay realistic in expectations, as the reports would rather serve keeping quality discussions in the public than allowing for quality comparisons [30].

### Medical errors

The questions of patient safety and risk management are critical for quality health care and therefore the issue of medical errors and malpractice is of direct relevance for quality management. The availability of data on medical errors is unsatisfactory, and the known numbers are considered the visible part of cases only with an assumed grey zone of unknown incidents [4,25,32]. According to the Robert Koch Institute suspected and documented medical errors in Germany count at estimated 40,000 per year with approximately 12,000 acknowledged damage claims [25]. Other authors suggest that approximately 5–10 per cent of all patients in hospitals experienced so-called undesired events, half of them classified as avoidable [24]. The majority of registered damage claims were related to surgical procedures, and hospital doctors were more often confronted with claims than office-based physicians. Explanations for these phenomena include the more anonymous patient-doctor relationship in hospitals and the easier recognition of errors in surgery [25,33].

From medical errors and especially from their causes conclusions can be drawn for the need of quality improvement measures. Causes can be identified at different levels: the individual health worker, the team, the health care facility or the whole health care system. While human mistakes more frequently generate damage, the system components combined with their complexity are more important [33]. The most common causes have been observed in organizational deficiencies, among which insufficient communication and coordination between providers rank highest (23 per cent). Structural factors of the German health system with its distinct sectors may trigger these coordination problems, manifest in weak management of the interfaces [25].

The prevention of medical errors and risk management need to be integral part of quality management. The role of internal quality management systems with their methods of continuous process and mistake analysis is the creation of a basis for risk management and thus improving patient safety [24]. The direct health work environment and especially the working conditions need to be taken into consideration for preventing errors and improving quality of care. High workloads, time constraints and weak interdisciplinary cooperation due to barriers between professional groups are contributing to adverse events in health care and undermine patient safety. While currently there is a hesitant attitude towards speaking about medical errors [32]. A new "error culture" would include the creation of protected platforms for discussion of adverse events and errors free from sanctions [25]. Admitting and accepting that adverse events and errors constitute a daily risk in health care would open the way for more active and systematic reporting systems. Lessons to be learnt from industry show that systematic (and sanction free) reporting of all unwanted events including those without consequences contributed significantly to the assessment of causes and resulted in better accident prevention [33].

### Patients and quality

In Germany research on patient concepts of quality of care has been emerging in recent years, similarly to quality research in general. The position of patients has been strengthened by legislation only since the health care reform 2000. While increasingly patient orientation is called for as one aspect of quality care, this demand conflicts with existing structures, goals, and a number of basic values, behaviour of professions and organizational procedures of institutions [25]. The recent concept of "shared decision making" requires a change in patterns of professional-patient-relationships. One major problem is the lack of patient adequate communication, which finds its roots in lack of communication skills of professionals, and in organizational and financial factors of the health system. Patient orientation requires citizen participation in health care development. This is a controversially discussed feature and experiences are described with mixed results. Nevertheless, citizen participation as a basic democratic principle is a value in itself and it can result in increased transparency [25]. Other countries are more advanced in user participation in quality management. In The Netherlands, for example, since the 1996 "Participation by Clients of Care Institutions Act" health care organizations are obliged to set up client councils and patients are enabled to influence the care provider's policy [34]. Patients need to be empowered for taking decisions by sound information. The declaration of patient rights is currently accompanied by strengthening independent patient counselling offices and by the new institutionalisation of patient representation in the Joint Federal Committee. As this is a quite new field in Germany experiences are not yet evaluated. Quality aspects of health care may be perceived and evaluated differently by patients than by professional actors in health care. The measuring of patient satisfaction through surveys is a common approach in quality management, while literature suggests major methodological challenges [36,37] and indicates the development phase of instruments assessing patient perception of quality [38-40]. Quality indicators on patient orientation have not yet been developed for the external hospital quality management [20] and patient relevant indicators have been suggested for introduction [19]. In general the awareness of the need of more patient orientation is rising while the implementation is in a beginning phase.

## Discussion

The overview of the history and recent developments of medical quality management in the German health system reveals an increased interest of policy makers in the monitoring and control of quality of care since the early nineties. Quality management became legally binding at times when concerns on the raising costs of health care scored high on the political agenda. While previous health care reforms nearly exclusively focussed on cost containment, quality management aspects gained momentum in the more recent reform legislation of 2000 and 2003.

However, one major critical point remains the prominently economical motivation of quality assurance measures in the German reform process. The KBV (Federal Association of GKV contracted doctors), for example, argues for the ambulatory medical care sector that the GMG 2003 would hide cost reduction interests behind quality assurance measures [41]. Others support the opinion that currently competition in the health system was cost-driven rather than quality or even quality-cost-relationship oriented [23,42]. International experiences further suggest that where the objectives of cost-control and quality were combined or when quality was used as argument for cost control, the success regarding improved quality has been least likely. Where cost-containment is the major motivation, the allocation of adequate resources for quality management may be insufficient [43]. Cost containment as the most prominent issue in health reform is confronted with the fact that quality management systems require considerable resources themselves. In Germany currently 20 million Euro are spent alone for the external quality management of hospitals, in major parts financed by the health insurance funds (and consequently by the contribution payers) [12].

A major challenge of quality managenent in Germany is the coordination of the various decision making bodies and expert committees requiring a structured cooperation of the bodies involved at the different levels. The coordination between state and federal level needs to be strengthened (information from an expert interview 2003 [4]). The list of institutional actors shows that there are many committees and institutions assigned for quality issues in health care. This carries the risk of redundant and duplicated work as well as the risk of intransparency [23]. The latest reform act has addressed this issue in concentrating a number of committees into one decision making body, the G-BA, while at the same time ensuring the participation of the major stakeholders.

The involvement of all stakeholders in the development of quality management strategies is an important factor for successful and sustainable implementation and adherence to quality oriented policies. The principle of self-governance in the German health system includes responsibilities of the providers for quality matters. Consequently the professional associations and representative bodies of doctors, insurance funds and the hospitals have played a major role in the past and continue to be key actors. Recently the Nursing Council has been involved as consultative partner in the quality management procedures and bodies. However, considering the quantitative and qualitative importance of nursing in health care and its close interrelation with medical care especially in hospital settings, it may be questioned why the Nursing Council does not have an equal right in decision making, meaning a vote, in the respective decision making bodies [19]. The involvement of the stakeholder group of patients is only in its very beginning.

It should be kept in mind that the quality discussion is not only a technical one but also driven by political and strategic motives of the various interest groups. Their controversial positions are reflected by the current literature [12,21,41,42]. The ongoing discourse between the parties concerned is a positive aspect. The consensus approach may not be the easiest way to process change but the most promising regarding the sustainability of achievements. Decisions on procedures, indicators and standards are better accepted if they are negotiated with those concerned. This includes the consideration that consensus agreements are most probably reflecting minimum standards rather than maximum options.

The slow pace of progress in the implementation of quality management may be partly due to these dialogue procedures, but it is also associated with reservations in introducing new patterns of cooperation between actors and institutions: lack of confidence, institutional competitive thinking ("silo thinking"), and the fear to loose territories are among the prevailing psychological barriers (information from interviews with experts, 2003 [4]). As the mandatory quality management is closely interlinked with the health care reform, this process altogether represents the dimension of a system's change where force fields (such as supportive and opposing interest groups), attitudes and behaviour play a significant role.

Among the structural factors hindering a coordinated quality management approach is the sectoral division of the health care. The weak management of the interfaces leads to a fragmented and often incoherent treatment of patients thus affecting quality of care. This could be addressed, as recommended by the patient representative for example, by developing quality indicators for hospitals that include the interface between hospital care, rehabilitation and post-hospital care [20]. Deficits in interdisciplinary and interprofessional communication and cooperation add to structural barriers. Mutual recognition, respect and enhanced room for constructive discussions would be an asset for quality improvement and patient safety [25]. This could also facilitate the coordination of guideline developments with regard to disease management programmes, which are frequently subject to conflicts between professional groups (information from an expert interview, 2003 [4].

From a public health point of view the question is pertinent whether quality assurance measures will show positive effects on the population health outcome. Another question would be to find out in which way quality goals, formulated by the self-governing partners, are linked to the national health goals. For example, a direct link between the national health goal "improving health competence, empowering patients" (the original wording reads: "Gesundheitliche Kompetenz erhöhen, Patientensouveränität stärken") and quality management issues is evident. Four objectives have been formulated within this goal: increasing transparency, developing capacity, strengthening patient rights, improving complaints procedures [44]. All these are of direct relevance for patient orientation in health care and quality management issues. Under the perspective of patient orientation the right for participation of patient representatives in decision making bodies such as the G-BA is a promising progress.

## Conclusion

Quality management in health care in Germany is on the move in a constructive direction. In the last 13 years considerable progress has been made. However, it is expected that the fully integrated implementation of quality management will take many years. Reasons for the long process can be seen in the complexity: It requires a long-term system change and sound methodological development of indicators suitable to the German health system. It further involves the education of the health professionals in quality management and assurance methods. This may also reduce some reservations of the various stakeholders vis-à-vis imposed quality approaches.

Quality management in Germany represents itself as a technical and professional issue. For the time being the aspect of patient orientation, one characteristic of the responsiveness of a health system [45], remains a weak part. The recent establishment of patient representatives participating in quality discussions opens the way for strengthening the patient orientation aspect. However, it may take a long time and requires resources to empower patient representatives to the degree of being accepted as real partners in the discussions.

Challenges in ensuring quality data, as well as the weak evidence on the effectiveness of hospital quality strategies have been addressed in the literature. The evaluation of the quality management efforts in terms of quality and efficiency is an emerging subject for the future.

The implementation of quality assurance measures in Germany since the nineties initiated a process, which also affects changes in the health system culture: Traditional reservations need to be overcome for new patterns of interdisciplinary and interprofessional cooperation. A new "error culture" needs an environment where adverse events and mistakes may be discussed and analysed constructively. The patient – professional relationship and communication patterns need to change for achieving better patient orientation and informed shared decision making.

## Abbreviations

AeZQ Ärztliches Zentrum für Qualität in der Medizin: Central Agency for Quality in Medicine

AOK Allgemeine Ortskrankenkasse: statutory regional/local health insurance fund

AQS Arbeitsgemeinschaft zur Förderung der Qualitätssicherung in der Medizin: Working Group for Promoting Quality Assurance in Medicine

AWMF Arbeitsgemeinschaft der Wissenschaftlichen Medizinischen Fachgesellschaften: Association of the Scientific Medical Societies

BAeK Bundesärztekammer: Federal Medical Chamber

BMGS Bundesministerium für Gesundheit und Soziales: Ministry of Health and Social Security

BQS Bundesgeschäftsstelle Qualitätssicherung: Federal Office for Quality Assurance

DemoProQM Demonstrationsprojekt Qualtitätsmanagement im Krankenhaus: Pilot Project Quality Management in Hospitals

DIMDI Deutsches Institut für Medizinische Dokumentation und Information: German Institute for Medical Documentation and Information

DKG Deutsche Krankenhausgesellschaft: German Hospital Federation

DNEbM Deutsches Netzwerk Evidenzbasierte Medizin: German Network of Evidence Based Medicine

DRG Diagnoses Related Groups

G-BA Gemeinsamer Bundesausschuss: Joint Federal Committee

GKV Gesetzliche Krankenversicherung: statutory health insurance

GMG Gesundheitsmodernisierungsgesetz: act on the modernization of the health care system

GQMG Gesellschaft für Qualitätsmanagement in der Gesundheitsversorgung: Association for Quality Management in Health Care

GRG Gesundheitsreformgesetz: health reform act

GSG Gesundheitsstrukturgesetz: law on reforming the health system structure

IQWiG Institut für Qualität und Wirtschaftlichkeit im Gesundheitswesen: Institute for Quality and Economy in Health Care

KBV Kassenärztliche Bundesvereinigung: Federal Association of GKV contracted medical doctors

KTQ^® ^Kooperation für Transparenz und Qualität im Krankenhaus: cooperation for transparency and quality in hospitals

KV Kassenärztliche Vereinigung: association of GKV contracted medical doctors

LQS Landesgeschäftsstelle Qualitätssicherung: regional, state based BQS office

MDK Medizinischer Dienst der Krankenkassen: medical review board of statutory health insurance funds

QEP^® ^Qualität und Entwicklung in Praxen: Quality and Development for Practice

QMK Qualitätsmodell Krankenhaus: project hospital quality model

SGB V Sozialgesetzbuch V: Social Welfare Code, Book (Volume) V

## Competing interests

The author(s) declare that they have no competing interests.

## Authors' contributions

All authors participated in the analysis and interpretation of the referenced papers. All authors read and approved the final manuscript.
